# A multilevel model for cardiovascular disease prevalence in the US and its application to micro area prevalence estimates

**DOI:** 10.1186/1476-072X-8-6

**Published:** 2009-01-30

**Authors:** Peter Congdon

**Affiliations:** 1 Department of Geography and Center for Statistics, Queen Mary University of London, Mile End Rd, London E1 4NS, UK

## Abstract

**Background:**

Estimates of disease prevalence for small areas are increasingly required for the allocation of health funds according to local need. Both individual level and geographic risk factors are likely to be relevant to explaining prevalence variations, and in turn relevant to the procedure for small area prevalence estimation. Prevalence estimates are of particular importance for major chronic illnesses such as cardiovascular disease.

**Methods:**

A multilevel prevalence model for cardiovascular outcomes is proposed that incorporates both survey information on patient risk factors and the effects of geographic location. The model is applied to derive micro area prevalence estimates, specifically estimates of cardiovascular disease for Zip Code Tabulation Areas in the USA. The model incorporates prevalence differentials by age, sex, ethnicity and educational attainment from the 2005 Behavioral Risk Factor Surveillance System survey. Influences of geographic context are modelled at both county and state level, with the county effects relating to poverty and urbanity. State level influences are modelled using a random effects approach that allows both for spatial correlation and spatial isolates.

**Results:**

To assess the importance of geographic variables, three types of model are compared: a model with person level variables only; a model with geographic effects that do not interact with person attributes; and a full model, allowing for state level random effects that differ by ethnicity. There is clear evidence that geographic effects improve statistical fit.

**Conclusion:**

Geographic variations in disease prevalence partly reflect the demographic composition of area populations. However, prevalence variations may also show distinct geographic 'contextual' effects. The present study demonstrates by formal modelling methods that improved explanation is obtained by allowing for distinct geographic effects (for counties and states) and for interaction between geographic and person variables. Thus an appropriate methodology to estimate prevalence at small area level should include geographic effects as well as person level demographic variables.

## Background

Estimates of prevalence of disease and health behaviours for different areas are increasingly required for the equitable allocation of health funds according to local need and to target interventions. As stressed by Bazos et al [1] community health need assessments are ideally based on locally disaggregated (i.e. small area) health status and disease prevalence information. To estimate prevalence in different small areas, a commonly adopted approach involves synthetic estimation whereby prevalence rates for demographic subgroups of the population are obtained (e.g. from national health surveys) and an indicative rate then obtained based on the demographic composition of each area. Thus prevalence of most health conditions varies considerably with age, and often also by sex and race: so a synthetic estimate may be obtained by using age, sex and race specific prevalence rates.

However, synthetic estimates of this kind do not take account of geographic context, exemplified by interactions between demographic risk factors and geographic location, or by independent effects of geographic variables (e.g. area poverty or urbanity-rurality) on prevalence that remain even after taking account of patient level risk factors. By contrast, the multilevel prevalence model for cardiovascular outcomes proposed here as a basis for small area prevalence estimates incorporates the modifying effects of geographic context as well as patient risk factors.

In the US, a number of population health surveys are carried out and provide cumulative evidence on CVD trends and epidemiology. Thus the National Health Interview Survey (NHIS) for 2005 estimated the prevalence of cardiovascular disease (CVD) at 68 million among adults aged 18 years and over in the US, which includes coronary heart disease, hypertension, stroke, angina pectoris or heart attack. The analysis here is concerned with a positive response to one or more of three questions included in the 2005 Behavioral Risk Factor Surveillance System (BRFSS) survey; these questions encompass the different forms of CVD, namely, had the subject ever been told by a health professional that they had experienced a heart attack, or told they had undergone a stroke, or told they had CHD or angina.

The epidemiology of these conditions differ to some degree, for example in terms of male-female differentials in prevalence and incidence [2], in trends through time [3], and in ethnic group differentials. However, for these and related conditions there is evidence for a role of geographic context, in terms of wide geographic disparities by region, state and urbanity [4-8]. In particular, there is evidence of direct effects of area variables after controlling for person level risk factors, and evidence of interactions between place and person variables. For example, Cubbin et al [9] report higher levels of hypertension and diabetes among African American women living in socioeconomically deprived neighborhoods as against African American women from more affluent neighborhoods, after allowing for individual-level socioeconomic status, while Halverson et al [10] report local clustering of excess CVD mortality after controlling for area population composition. As for place-person interactions, Barnett et al [6] and Casper et al [11] report that ethnic disparities in CHD mortality vary by area of residence.

The prevalence model and small area prevalence estimates described here are based on around 336,000 survey responses, and on a regression analysis relating CVD status both to individual level risk factors and to county level measures of poverty and urban-rural status. The analysis further adjusts for differentiation at US state level in the impact of ethnicity on prevalence. Thus adjustment for geographic context is much more comprehensive than is possible using disease status data from the Health Survey for England where only broad regional identifiers are available – an example being the work of Congdon [12] on CHD prevalence. One goal of the analysis here is to develop prevalence estimates for micro areas, namely 32000 ZIP Code Tabulation Areas (ZCTAs) for which certain population tabulations are provided by the US Census Bureau [13]. Inclusion in the prevalence model of patient risk categories such as gender and ethnicity (and interactions between them) therefore requires that such categories are available in these tabulations for micro area populations.

## Methods

The regression model for prevalence includes person level attributes (age, gender, ethnicity, education level) that are known to have significant CVD risk gradients. A pronounced gradient in CVD prevalence by age is reported by Neyer et al [14]; thus the MI rate among 18–44 year olds is 0.8%, among 45–64 year olds is 4.8% and among the over 65s is 12.9%. In terms of the main ethnic groups in the US (white non-hispanic, black, hispanic, other) elevated CVD mortality and morbidity for nonwhite groups are reported by Barnett et al [6] and Caspar et al [11], though ethnic differentials may to some degree express socioeconomic disadvantage. Certain subgroups such as black females, have more clearly elevated CVD prevalence [15]. As to education level, Neyer et al [14] report that prevalence of one or more of an MI history or a CHD/angina history decreases with educational attainment: of persons with less than a high school diploma, 9.8% report a history of one or more of the conditions, nearly twice the proportion (5%) among college graduates. Education is interrelated with issues such as linguistic competence and health literacy that affect health status [16], and with health insurance [17].

### Methods: Translating Survey Model to Small Area Prevalence Estimates

However, to permit small area (ZCTA) prevalence estimation, inclusion of risk variables (and interactions between them) in the regression model is subject to the constraint that such variables are available both in the BRFSS and in tabulations for ZCTA populations. So an interaction between risk factors requires a matching cross-tabulation in the ZCTA population. Impacts of age group, gender and ethnic group are straightforward to include since they are available as BRFSS variables and in a ZCTA level cross-tabulation of adult populations by gender, ethnicity, and quinquennial age. For particular gender-ethnic-age subgroups, parameters from the survey model (e.g. relative risk for white males aged 65–69) can then be applied to the ZCTA sub-population.

For other person level variables (e.g. education, marital status), either primary ZCTA tabulations are available from the 2000 census, or a restricted cross tabulation (e.g. adult population by education, ethnicity and gender in US census tabulation P148), but not tabulations involving cross-hatching against all other risk factors. A small area prevalence adjustment can be applied only for the main effect of such variables, or for a partial interaction. Thus the BRFSS regression models include gender-education effects, and so gradients in CVD relative risk can be applied to ZCTA male and female adult populations subdivided by education level. Gender-education-ethnic interactions are not adopted as the relevant ZCTA cross tabulation often includes very small numbers.

### Methods: The Prevalence Model

The regression involves 129 thousand male and 207 thousand female respondents, and is confined to adults aged 18 and over. Separate regressions are carried out for males and females, in view of evidence of gender effect modification over a range of risk variables [18]. The regression also takes account of varying survey weights *w *for different respondents to account for differential response between demographic categories and for different sampling rates in different US states. The detailed derivation of weights is discussed in CDC [19] and is based on the inverse of the sampling fraction in each area stratum and age-by-race-by-gender category.

Let *y *= 1 if a subject reports a particular CVD symptom, with *y *= 0 otherwise, and denote *p *as the probability that a respondent reports a symptom. Then a weighted likelihood [20] over subjects *i *and gender *r *(*r *= 1 for males, 2 for females) is used, giving greater weight to undersampled demographic categories or areas, namely

(1) $$\prod_{i}p_{ir}^{y_{ir}w_{ir}}{\left(1-p_{ir}\right)}^{w_{ir}(1-y_{ir})}.$$

To facilitate a relative risk interpretation for parameters a log link is used in the binary regression [21] – see Appendix 1 for model details. In Winbugs this requires (a) a model regression statement linking *log*($p_{ir}^{*}$) to risk factor covariates and any random effects and (b) a statement selecting the minimum of 1 and $p_{ir}^{*}$ as the actual probability *p*_*ir *_that *y*_*ir *_= 1. The occurrence of values $p_{ir}^{*}$ > 1 was confined within the first hundred or so MCMC iterations (depending on how close the starting parameter values are to the posterior means), and thereafter convergence was straightforward.

Three types of regression model are applied in order to assess geographic effects. The first baseline model (model 1) includes only person level risk variables. It allows first for differential risks of each CVD symptom for black, hispanic and other ethnic groups as against whites as the reference category. Second, it allows differential risk according to education attainment with categories 1 = never attended, elementary only, or some high school; 2 = high school graduate; 3 = some college or technical school; 4 = college graduate (with level 1 as reference category for statistical estimation). Finally, since age gradients are known to vary by ethnic group, differential risks are assumed specific to combinations of age group (12 levels) and the four ethnic groups; the age bands are 18–24,25–29,30–34,..,70–74, and 75+.

The second type of model (model 2) includes geographic effects but without any interaction between area and person attributes (except for gender). Although prevalence is to be estimated for ZCTAs, the ZCTA of residence for BRFSS respondents is not available for confidentiality reasons. However, county and state of residence are provided, and one may model their impact on CVD prevalence. Since there are over 3000 US counties, some counties are sparsely represented in the survey, and so random effects at this level are not adopted. However, county level variables are used as predictors, these being the 2005 percent of population in poverty and a category variable, namely the 9 category rural-urban continuum coding [22] – see Table 1.

**Table 1:** Categorisation of Counties by Rural-Urban Continuum*

***Code***	***Description***	***Metropolitan Type***
1	Counties in metro areas of 1 million population or more	Metropolitan
2	Counties in metro areas of 250,000 to 1 million	Metropolitan
3	Counties in metro areas of fewer than 250,000	Metropolitan
4	Urban population of 20,000 or more, adjacent to a metropolitan area	Non-metro
5	Urban population of 20,000 or more, not adjacent to a metropolitan area	Non-metro
6	Urban population of 2,500 to 19,999, adjacent to a metropolitan area	Non-metro
7	Urban population of 2,500 to 19,999, not adjacent to a metropolitan area	Non-metro
8	Completely rural or less than 2,500 urban population, adjacent to a metropolitan area	Non-metro
9	Completely rural or less than 2,500 urban population, not adjacent to a metropolitan area	Non-metro

* see

Many geographic influences may be unobserved (e.g. various environmental and health behavioral influences) and these are represented in the second and third models by state level random effects. These are modelled using a random effects approach (see Appendix 2) that allows both for spatial correlation between effects for contiguous states and for the presence of spatially isolated states. It is sensible to allow unobserved state influences to be spatially correlated to reflect smoothly varying risk factors in space [23]. However, application of conditional autoregressive spatial schemes [24], with spatial interaction typically based on contiguity of areas, is complicated by the presence of two spatially isolated states (Alaska, Hawaii). A different approach based on Congdon [25] is applied instead, which allows for varying strength in spatial clustering over the mainland states and also encompasses spatial isolates. In model 2, effects of county poverty and urbanity are included together with random effects for the 51 states.

The third model (model 3) allows for area-person interactions, in that state random effects are taken to be ethnicity specific. Differentiation of area effects by ethnicity reflects epidemiological evidence such as that from Casper et al [11] that CVD mortality and prevalence disparities between ethnic groups vary by place of residence. Let *C*_*i *_and *S*_*i *_respectively denote the county and state in which subject *i *is resident. Let *r*_*i *_denote a subject's gender, *g*_*i *_denote their ethnic group, *x*_*i *_denote their age group, and *e*_*i *_denote their education level. Then the prevalence probability is specified under the full model as

(2) *p*[*r*_*i*_, *g*_*i*_, *e*_*i*_, *x*_*i*_,*C*_*i*_, *S*_*i*_] = *exp*(*α*[*r*_*i*_] + *β*[*r*_*i*_, *g*_*i*_] + *η*[*r*_*i*_, *e*_*i*_] + *γ*[*r*_*i*_, *x*_*i*_, *g*_*i*_] + *κ*[*r*_*i*_]*Pov*[*C*_*i*_] + *δ*[*r*_*i*_, *U*[*C*_*i*_]] + *w*[*r*_*i*_, *S*_*i*_, *g*_*i*_]),

where *α*_*r *_are gender specific intercepts measuring the overall prevalence level, the *β*_*rg *_parameters measure varying prevalence by ethnicity, the *η*_*re *_terms measure varying prevalence by education, the *γ*_*rxg *_measure ethnic specific age gradients, *κ*_*r *_is the coefficient for county poverty, the *δ*_*ru *_terms reflect the effect of different categories *U *in the rural-urban continuum, and the *w*_*rsg *_terms are state random effects specific for ethnic group. County poverty rates (for all ages in 2005) are expressed as proportions and range from 0.025 to 0.51, and are centred around the average poverty rate.

### Methods: ZCTA Prevalence Rates

To translate the prevalence model parameters into ZCTA level estimates requires categorisations of the ZCTA populations that match the survey derived individual and geographic risk factors used in the prevalence model. The goal is to obtain ZCTA age-sex-ethnic prevalence rates (and case totals) that reflect not only demographic gradients, but also reflect the impact that the location and socioeconomic character of the ZCTA have on prevalence. Among important socioeconomic influences on disease (including CVD) that are available for ZCTAs in 2000 Census tabulations are education, income, poverty status, and household tenure.

Here education is used as a socioeconomic measure of small area populations because of established CVD prevalence gradients by education level [14], and because it is available both as a BRFSS survey question and in ZCTA census tabulations. Education has been used as a measure of socioeconomic status in other area health studies [26]. Essentially the age-sex-ethnic rates obtained from the survey prevalence model (for the reference education group) are adjusted according to a sex-specific education effect that is also estimated in the model.

Let *C*_*j *_and *S*_*j *_respectively denote the county and state in which ZCTA *j *is located. Let *r *denote gender, *g *denote ethnic group and *x *denote age group. Then given a particular county *C*_*j *_and state of residence *S*_*j*_, prevalence rates for ZCTA *j *specific to age-sex-ethnic group, but unadjusted for that ZCTA's education mix, are obtained from the full model as

(3) *p*[*j*, *r*, *x*, *g*] = *exp*(*α*[*r*] + *β*[*r*, *g*] + *γ*[*r*, *x*, *g*] + *κ*[*r*]*Pov*[*C*_*j*_] + *δ*[*r*, *U*[*C*_*j*_]] + *w*[*r*, *S*_*j*_, *g*]).

This is the model for the reference education group (namely, the group with less than high school education). As described in Appendix 1, the *β *and *γ *parameters represent ethnic and age-ethnic effects for gender *r*; the parameters *κ *and *δ *represent county poverty and urban-rural effects, and the *w *parameters are state level random effects.

To take account of the impact on CVD prevalence of education attainment mix, let *π*[*j, r, e*] be the 2000 census data relative proportions at education level *e *in each gender's adult population in ZCTA *j*. Also let

(4) *λ*[*r, e*] = *exp*(*η *[*r, e*])

be the survey model estimate of CVD relative risk at education level *e *after controlling for age, ethnicity and geographic effects (county and state effects). The composite relative risk associated with the educational mix in ZCTA *j *can be represented as a weighted total of the relative risks for each education level, namely

(5) $$L[j,r]=\sum_{e}\pi [j,r,e]\lambda [r,e].$$

Finally, age-sex-ethnic prevalence rates *p*_*a*_[*j, r, x, g*] in ZCTA *j *adjusted for its education mix are obtained as

(6) *p*_*a*_[*j, r, x, g*] = *p*[*j, r, x, g*]*L*[*j, r*].

## Results

Estimation of the three models follows the Bayesian method, whereby pre-existing knowledge regarding parameters is expressed in prior densities, and updated or posterior knowledge is obtained by combining the prior densities with the likelihood (1) of the observed data. Estimation uses iterative Monte Carlo Markov Chain sampling methods [27], as provided in the WINBUGS program [28]. Goodness of fit is assessed by the Deviance Information Criterion or DIC [29], whereby the average deviance is adjusted to account for model complexity. The DIC is the average deviance plus the complexity, with lower DICs representing better fit. Summaries of parameters (means and 95% intervals) are based on the second halves of two chain runs of 5000 iterations, with dispersed initial values. Convergence was achieved in all models using Brooks-Gelman-Rubin criteria [30].

Table 2 summarises the fit of the models, while Tables 3 and 4 show gender-specific es-timates of the parameters {*α*_*r*_, *β*_*rg*_, *η*_*re*_, *κ*_*r*_, *δ*_*ru*_} from the three models. The DIC criteria in Table 2 show a gain in introducing geographic contextual variables (model 2 vs model 1), and a clear gain also in making state random effects specific to ethnic groups (model 3 vs model 2).

**Table 2:** Summary of Model Fit

		**Average** **Deviance**	**Complexity**	**DIC**
Males	Model 1	64490	33	64523
	Model 2	64400	73	64473
	Model 3	64182	105	64287
Females	Model 1	91157	41	91198
	Model 2	90858	86	90944
	Model 3	90567	107	90674

**Table 3:** Cardiovascular Prevalence Models 1 to 3, Parameter Estimates for Males

	**Model 1**				**Model 2**				**Model 3**			
	**Mean**	**2.5%**	**97.5%**	**Rel've Risk**	**Mean**	**2.5%**	**97.5%**	**Rel've Risk**	**Mean**	**2.5%**	**97.5%**	**Rel've Risk**
*α*	-2.49	-2.53	-2.45		-2.51	-2.72	-2.43		-2.45	-2.54	-2.41	
Ethnic Coefficients (log relative risk)*												
*β*_11_	0.00			1.00	0.00			1.00	0.00			1.00
*β*_12_	0.06	0.00	0.14	1.07	-0.05	-0.12	0.03	0.95	-0.08	-0.17	-0.01	0.92
*β*_13_	0.04	-0.01	0.08	1.04	0.08	0.00	0.20	1.09	0.01	-0.11	0.10	1.01
*β*_14_	0.25	0.18	0.31	1.28	0.17	0.08	0.24	1.18	0.21	0.10	0.32	1.24
Education Coefficients (log relative risk)**												
*η*_11_	0.00			1.00	0.00			1.00	0.00			1.00
*η*_12_	-0.22	-0.26	-0.17	0.81	-0.20	-0.24	-0.15	0.82	-0.21	-0.26	-0.15	0.81
*η*_13_	-0.24	-0.29	-0.19	0.78	-0.22	-0.27	-0.16	0.80	-0.24	-0.29	-0.17	0.79
*η*_14_	-0.56	-0.60	-0.51	0.57	-0.53	-0.58	-0.47	0.59	-0.55	-0.59	-0.48	0.58
County Effects												
*κ*_1 _(County poverty)***					0.48	0.18	0.83	1.11	0.46	0.17	0.78	1.10
*δ*_1 _Parameters (Urbanity)												
Metro > 1 m					0.00			1.00	0.00			1.00
Metro, 250th-1m					-0.03	-0.09	0.02	0.97	-0.02	-0.06	0.02	0.98
Metro < 250th					0.02	-0.04	0.09	1.02	0.02	-0.03	0.07	1.02
Urban > 20th, adj Metro					-0.08	-0.15	-0.01	0.92	-0.07	-0.14	-0.01	0.93
Urban > 20th, not adj Metro					-0.12	-0.23	-0.01	0.88	-0.11	-0.20	0.00	0.89
Urban 2.5–20th, adj Metro					-0.02	-0.09	0.06	0.98	0.00	-0.07	0.06	1.00
Urban 2.5–20th, not adj Metro					0.04	-0.04	0.12	1.04	0.04	-0.03	0.12	1.04
Rural or < 2,5th, adj Metro					0.01	-0.09	0.16	1.01	0.03	-0.08	0.14	1.03
Rural or < 2,5th, not adj Metro					0.01	-0.14	0.19	1.01	0.04	-0.07	0.13	1.04
State Spatial Effects												
*λ*_a_, Average Spatial Dependence					0.57	0.27	0.81		0.38	0.15	0.62	
*τ*_w _Overall Spatial Variance (Model 2)					0.011	0.005	0.025					
*φ*_11 _Spatial Variance, Wh (Model 3)									0.010	0.005	0.017	
*φ*_22 _Spatial Variance, Blk (Model 3)									0.077	0.033	0.167	
*φ*_33 _Spatial Variance, Hisp (Model 3)									0.041	0.019	0.078	
*φ*_44 _Spatial Variance, Oth (Model 3)									0.021	0.009	0.045	

* 1 White; 2 Black; 3 Hispanic; 4 Other

** 1 No school, elementary only, or some high school without graduating; 2 High school graduate; 3 Some college; 4 College Graduate

*** Relative Risks for County Poverty Compares Risks at 5th and 95th percentiles of 2005 all age poverty rate

**Table 4:** Cardiovascular Prevalence Models 1 to 3, Parameter Estimates for Females

	**Model 1**				**Model 2**				**Model 3**			
	**Mean**	**2.5%**	**97.5%**	**Rel've Risk**	**Mean**	**2.5%**	**97.5%**	**Rel've Risk**	**Mean**	**2.5%**	**97.5%**	**Rel've Risk**
*α*	-2.58	-2.63	-2.55		-2.67	-2.72	-2.61		-2.66	-2.72	-2.61	
Ethnic Coefficients (log relative risk)*												
β_21_	0.00			1.00	0.00			1.00	0.00			1.00
β_22_	0.38	0.33	0.43	1.46	0.35	0.27	0.43	1.42	0.32	0.25	0.41	1.38
β_23_	0.10	0.04	0.16	1.11	0.11	0.04	0.16	1.11	-0.01	-0.12	0.06	0.99
β_24_	0.34	0.28	0.40	1.41	0.37	0.29	0.43	1.44	0.48	0.42	0.56	1.62
Education Coefficients (log relative risk)**												
*η*_21_	0.00			1.00	0.00			1.00	0.00			1.00
*η*_22_	-0.41	-0.45	-0.38	0.66	-0.38	-0.41	-0.34	0.69	-0.37	-0.40	-0.32	0.69
*η*_23_	-0.47	-0.51	-0.42	0.63	-0.43	-0.47	-0.39	0.65	-0.42	-0.47	-0.38	0.65
*η*_24_	-0.99	-1.03	-0.94	0.37	-0.93	-0.98	-0.88	0.39	-0.92	-0.97	-0.87	0.40
*County Effects*												
*κ*_2 _(County poverty)***					0.80	0.48	1.10	1.18	0.83	0.51	1.17	1.19
*δ*_2 _Parameters (Urbanity)												
Metro > 1 m					0.00			1.00	0.00			1.00
Metro, 250th-1m					0.01	-0.03	0.05	1.01	0.02	-0.02	0.06	1.02
Metro < 250th					0.07	0.03	0.12	1.07	0.06	0.01	0.11	1.06
Urban > 20th, adj Metro					0.07	-0.01	0.12	1.07	0.08	0.01	0.16	1.09
Urban > 20th, not adj Metro					-0.03	-0.14	0.09	0.97	0.01	-0.06	0.10	1.01
Urban 2.5–20th, adj Metro					0.10	0.05	0.15	1.11	0.10	0.05	0.15	1.10
Urban 2.5–20th, not adj Metro					0.04	-0.03	0.11	1.04	0.05	-0.02	0.13	1.05
Rural or < 2,5th, adj Metro					0.04	-0.13	0.17	1.04	0.04	-0.07	0.18	1.04
Rural or < 2,5th, not adj Metro					0.01	-0.07	0.10	1.01	0.01	-0.12	0.14	1.01
*State Spatial Effects*												
*λ*_*a*_, Average Spatial Dependence					0.59	0.24	0.86		0.28	0.11	0.57	
*τ*_*w *_Overall Spatial Variance (Model 2)					0.031	0.016	0.054					
*φ*_11 _Spatial Variance, Wh (Model 3)									0.023	0.011	0.049	
*φ*_22 _Spatial Variance, Blk (Model 3)									0.028	0.012	0.052	
*φ*_33 _Spatial Variance, Hisp (Model 3)									0.064	0.033	0.117	
*φ*_44 _Spatial Variance, Oth (Model 3)									0.078	0.046	0.132	

* 1 White; 2 Black; 3 Hispanic; 4 Other

** 1 No school, elementary only, or some high school without graduating; 2 High school graduate; 3 Some college; 4 College Graduate

*** Relative Risks for County Poverty Compares Risks at 5th and 95th percentiles of 2005 all age poverty rate

### Results: Person Level Attributes

**Figure 1:**
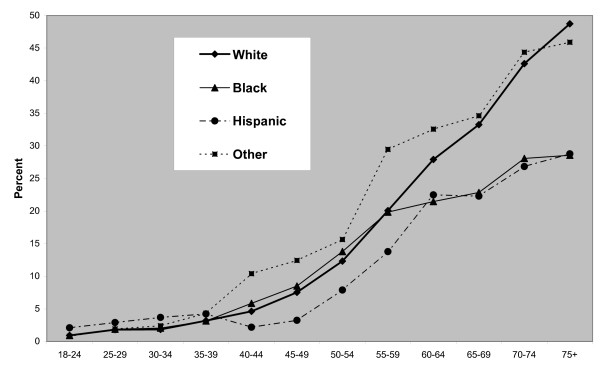
**Ethnic specific age gradients, males**.

**Figure 2:**
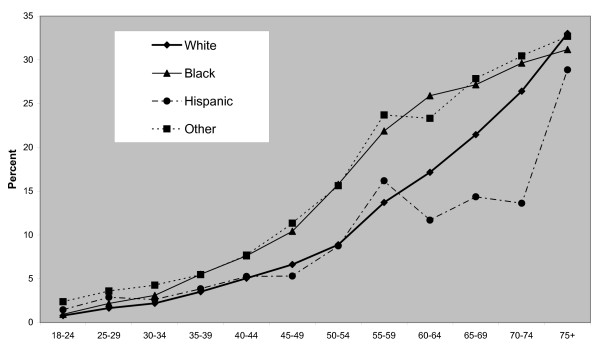
**Ethnic specific age gradients, females**.

In terms of person-level attributes, it can be seen from Tables 3 and 4 that there is a steeper educational gradient for females than males. In model 3, the relative risk for female college graduates is *exp*(*η*_24_) = 0.40 is under a half that of the first education category, those with limited education (elementary education only or did not graduate from high school). Black females also show excess CVD risk (an excess that remains after controlling for socioeconomic and geographic effects), whereas black males do not. However, both males and females in the other ethnic group have elevated risk. The ethnic specific age gradients for males (*γ*_1*xg*_) and for females (*γ*_2*xg*_) under model 3 are shown in Figures 1 and 2. The age gradients are presented in the form

(7) *p*_*rxg *_= *exp*(*α*_*r *_+ *β*_*rg *_+ *γ*_*rxg*_),

namely probabilities of CVD caseness by gender, age and ethnicity at reference levels of education and county urbanity and average county poverty. There are cross-over effects between black and white males with higher rates for black males up to early old age, and but lower rates thereafter. This reflects a wider finding that blacks "experience heart disease and die of heart-related problems at earlier ages than whites" [31]. For black females prevalence rates exceed those among white females except among the very old.

Probabilities of CVD by gender, age, ethnicity and education at reference levels of county urbanity and average county poverty are obtained as

(8) *p*_*rxge *_= *exp*(*α*_*r *_+ *β*_*rg *_+ *η*_*re *_+ *γ*_*rxg*_).

The overall age adjusted prevalence *p*_*rge *_for ethnic groups *g *at education level *e *may be obtained by using age weights *w*_*x *_for a standard population (e.g. the European Standard Population), namely

(9) $$p_{rge}=\sum_{x}w_{x}p_{rxge}.$$

Table 5 contains posterior summaries (expressed as percents CVD caseness) of the *p*_*rge *_over the four ethnic groups and four education levels. The widest contrast is among women, exemplified by the rates for white, college-educated women (mean prevalence of 3.0%), as opposed to women of other ethnicity with limited education (mean prevalence of 11.8%). The stronger effect of education on female risk means that the male to female risk ratio is higher for college graduates than those with lesser education.

**Table 5:** Posterior Mean Cardiovascular Prevalence Rates (Percents) by Gender, Ethnicity, and Education

		**Males**			**Females**			
**Ethnicity**	**Education**	**Mean**	**2.5%**	**97.5%**	**Mean**	**2.5%**	**97.5%**	**Male-Female Risk Ratio**
White	No High School	9.8	9.0	10.5	7.6	7.5	7.7	1.28
	High Sch Graduate	8.2	7.8	8.7	5.4	5.2	5.5	1.53
	Some College	8.1	7.6	8.6	5.1	5.0	5.3	1.59
	College Graduate	5.9	5.6	6.2	3.0	3.0	3.1	1.94
Black	No High School	8.7	8.2	9.4	10.3	9.9	11.0	0.85
	High Sch Graduate	7.3	6.9	7.8	7.2	7.0	7.6	1.01
	Some College	7.2	6.8	7.6	6.8	6.6	7.3	1.05
	College Graduate	5.2	4.9	5.6	4.1	3.8	4.4	1.28
Hisp	No High School	8.4	7.9	9.1	6.5	6.2	6.7	1.30
	High Sch Graduate	7.1	6.6	7.6	4.5	4.3	4.8	1.55
	Some College	7.0	6.5	7.5	4.3	4.1	4.5	1.62
	College Graduate	5.1	4.7	5.5	2.6	2.5	2.7	1.97
Other	No High School	13.0	12.3	13.8	11.8	11.5	12.1	1.10
	High Sch Graduate	10.9	10.4	11.6	8.3	8.1	8.5	1.31
	Some College	10.7	10.2	11.3	7.8	7.7	8.0	1.36
	College Graduate	7.8	7.4	8.4	4.7	4.5	4.9	1.66

### Results: Geographic Variables

Tables 3 and 4 show that the county poverty effect is more pronounced for female than male CVD caseness. Whereas all county poverty effects are significant, many of the coefficients for the county urban-rural category are not significant. Significance of urban-rural category differs whether model 2 or model 3 is considered, and also differs to some extent by gender. Under model 3, male risks are significantly low in the non-metropolitan category "urban population with over 20 thousand or more, adjacent to a metropolitan area", while under model 2, significantly lower risk prevails in both categories of "urban population with over 20 thousand or more". These may be interpreted as categories intermediate between highly metropolitan and highly rural settings, and the lower risks there fit with the view of Ingram & Franco [32] that metropolitan and rural areas tend to have worse health than intermediate area types. However, for females under model 3, counties in smaller metropolitan areas, as well as those with urban populations over 2500 and adjacent to a metropolitan area, have a significantly elevated risk. The absence of clear patterns may be because the association between urban status and health is linked to the uneven distribution of poverty in the US, which tends to be disproportionately concentrated in metropolitan centres as well as in some rural areas [33]. So rural-urban prevalence gradients may be attenuated once poverty levels are controlled for.

State level random effects are included in both models 2 and 3 (see Appendix 2). A summary expression of unobserved state level influences applicable across all ethnic groups is obtainable from the additive person and area effects model 2 – see Table 6. These are residual relative risks in the form

**Table 6:** Residual State Effects (Relative Risk) Model 2 Significantly high in bold, significantly low in bold and italicised

		**Males**			**Females**		
Regional Division	State	Mean	2.5%	97.5%	Mean	2.5%	97.5%
East North Central	Illinois	1.09	0.99	1.23	0.96	0.89	1.04
East North Central	Indiana	**1.10**	**1.03**	**1.23**	1.00	0.91	1.11
East North Central	Michigan	1.06	0.96	1.16	**1.10**	**1.01**	**1.20**
East North Central	Ohio	0.97	0.90	1.10	1.05	0.97	1.15
East North Central	Wisconsin	0.98	0.88	1.06	***0.82***	***0.70***	***0.94***
East South Central	Alabama	1.11	0.99	1.26	1.04	0.93	1.16
East South Central	Kentucky	**1.10**	**1.02**	**1.22**	**1.18**	**1.07**	**1.30**
East South Central	Mississippi	1.07	0.96	1.20	**1.14**	**1.00**	**1.27**
East South Central	Tennessee	1.06	0.97	1.19	**1.16**	**1.07**	**1.28**
Middle Atalantic	New Jersey	0.97	0.90	1.07	0.98	0.89	1.06
Middle Atalantic	New York	1.00	0.94	1.09	***0.90***	***0.83***	***0.97***
Middle Atalantic	Pennsylvania	1.01	0.94	1.12	0.99	0.92	1.06
Mountain	Arizona	1.02	0.96	1.11	0.96	0.88	1.03
Mountain	Colorado	***0.88***	***0.79***	***0.96***	***0.91***	***0.79***	***0.99***
Mountain	Idaho	0.99	0.91	1.10	0.99	0.87	1.13
Mountain	Montana	0.92	0.82	1.01	1.02	0.88	1.19
Mountain	Nevada	0.99	0.91	1.08	1.15	0.97	1.31
Mountain	New Mexico	0.96	0.84	1.10	0.89	0.77	1.03
Mountain	Utah	0.98	0.90	1.06	0.93	0.77	1.05
Mountain	Wyoming	0.96	0.86	1.05	0.96	0.81	1.13
New England	Connecticut	0.99	0.89	1.09	0.92	0.83	1.02
New England	Maine	0.96	0.84	1.12	0.98	0.82	1.16
New England	Massachusetts	1.02	0.95	1.09	0.92	0.80	1.04
New England	New Hampshire	0.99	0.90	1.10	1.06	0.89	1.28
New England	Rhode Island	0.96	0.83	1.12	0.89	0.77	1.07
New England	Vermont	1.02	0.92	1.17	0.89	0.73	1.12
Pacific	California	***0.91***	***0.85***	***0.97***	1.04	0.98	1.11
Pacific	Oregon	0.96	0.88	1.06	0.96	0.85	1.08
Pacific	Washington	0.96	0.88	1.06	0.93	0.83	1.03
Pacific	Alaska	1.00	0.80	1.25	1.00	0.71	1.42
Pacific	Hawaii	0.94	0.83	1.09	***0.83***	***0.67***	***0.99***
South Atlantic	Delaware	1.03	0.95	1.19	1.02	0.88	1.17
South Atlantic	District of Columbia	1.00	0.88	1.15	0.94	0.77	1.12
South Atlantic	Florida	1.06	0.99	1.17	**1.21**	**1.13**	**1.30**
South Atlantic	Georgia	1.04	0.94	1.14	0.96	0.87	1.04
South Atlantic	Maryland	0.99	0.90	1.09	0.98	0.87	1.09
South Atlantic	North Carolina	1.05	0.96	1.16	1.01	0.93	1.10
South Atlantic	South Carolina	1.03	0.92	1.12	0.94	0.82	1.05
South Atlantic	Virginia	**1.10**	**1.02**	**1.21**	0.99	0.91	1.10
South Atlantic	West Virginia	1.06	0.97	1.21	**1.40**	**1.26**	**1.55**
West North Central	Iowa	1.00	0.91	1.08	0.95	0.83	1.06
West North Central	Kansas	1.02	0.93	1.11	1.01	0.87	1.15
West North Central	Minnesota	0.93	0.82	1.01	***0.86***	***0.78***	***0.97***
West North Central	Missouri	1.06	0.99	1.17	1.09	0.98	1.19
West North Central	Nebraska	0.98	0.87	1.07	0.92	0.80	1.03
West North Central	North Dakota	0.96	0.85	1.08	0.95	0.82	1.08
West North Central	South Dakota	0.97	0.84	1.06	0.99	0.85	1.15
West South Central	Arkansas	1.02	0.89	1.19	1.04	0.91	1.17
West South Central	Louisiana	**1.10**	**1.01**	**1.22**	1.02	0.93	1.16
West South Central	Oklahoma	1.07	0.95	1.20	1.04	0.95	1.15
West South Central	Texas	1.02	0.94	1.17	**1.15**	**1.06**	**1.24**

(10) *ρ*_*rs *_= *exp*(*w*_*rs*_),

over states *s*, and amount to residual effects after controlling for the age, ethnic and educational composition of populations, and also for county poverty and urbanity. High residual relative risks, namely those significantly exceeding 1 (in the sense that the 95% credible interval is confined to values over 1) tend to occur in the South East and South of the US. For males elevated unexplained risks are present in Indiana, Kentucky, Louisiana and Virginia, and for females in Kentucky, Mississippi, Tennessee, Texas and West Virginia. Significantly low relative risks, those significantly under 1, occur for males in California and Colorado, and for females in Colorado, Minnesota, New York and Hawaii.

When residual state effects are made ethnic-specific in model 3, there are clear contrasts in variability between ethnic groups (see the spatial variance estimates in Tables 3 and 4). For males, there is greater variability in black and hispanic unexplained relative risk than for non-hispanic whites, while for females variability is greatest for hispanic and other ethnicities. To summarise the relative risk patterns, and in particular the location of states with two or more *ρ*_*rsg*_= *exp*(*w*_*rsg*_) significantly above 1, the nine Census Bureau Regional Divisions (listed in Table 6) are used to categorise the states (Table 7). There are consistent patterns, with multiple elevated residual effects tending to occur in the South (South Atlantic, East South Central) and East North Central divisions; this pattern shows similarities with that found by studies such as [8], though here the pattern is one that persists after controlling for important person and county risk factors.

**Table 7:** States with Elevated Residual Risk (95% interval exceeding 1) according to Census Division.

	**Males**			**Females**		
Division	Zero	One	Two	Zero	One	Two
East North Central	1	3	1	0	3	2
East South Central	1	2	1	0	2	2
Middle Atalantic	3	0	0	3	0	0
Mountain	7	1	0	7	0	1
New England	6	0	0	4	2	0
Pacific	5	0	0	3	1	1
South Atlantic	5	2	2	7	1	1
West North Central	6	1	0	5	2	0
West South Central	3	0	1	3	1	0
Total	37	9	5	32	12	7

Number of states with elevated residual RR

### Results: ZCTA Prevalence Estimates

As discussed above, the model provides estimates of *p*_*a*_[*j, r, x, g*] for approximately 32 thousand ZCTAs in 51 states. These are gender-ethnic-age prevalence rates adjusted for the education mix of each ZCTA. Summary ZCTA prevalence rates for gender-ethnic combinations may then be obtained by applying standard population age weights *w*_*x*_, namely

(11) $$p_{a}[j,r,g]=\sum_{x}w_{x}p_{a}[j,r,x,g].$$

Implications for prevalence levels and prevalence inequalities by state or county can then be assessed by considering relevant subsets of the gender-ethnic rates. Being able to assess small area inequality in health is important in health needs assessment [1].

Thus Table 8 presents female prevalence levels for the three main ethnic groups across the 51 states, obtained by averaging *p*_*a*_[*j*, 2, *g*] within states. Also shown are within state variances and ranges of the ZCTA prevalences. Prevalence levels and within state variability both tend to be higher in southern states such as Alabama, Kentucky, Louisiana, Mississippi, Texas and West Virginia.

**Table 8:** ZCTA Cardiovascular Prevalence Estimates for Females: State Averages and Variability by Ethnicity

	**White Females**			**Black Females**			**Hispanic Females**		
	**Average**	**Variance**	**Range**	**Average**	**Variance**	**Range**	**Average**	**Variance**	**Range**
Alabama	8.26	1.22	7.02	9.21	1.51	7.83	7.14	0.91	6.06
Arizona	6.70	1.30	6.08	10.41	3.14	9.48	7.55	1.65	6.86
Arkansas	8.42	0.75	5.86	10.58	1.18	7.36	7.20	0.55	5.01
California	6.06	0.79	4.62	9.91	2.12	7.54	8.59	1.59	6.54
Colorado	5.41	0.52	3.63	7.96	1.12	5.37	6.39	0.72	4.31
Connecticut	4.98	0.27	2.70	6.73	0.49	3.65	5.04	0.27	2.73
Delaware	7.70	0.70	3.37	9.02	0.95	3.92	7.96	0.74	3.46
Distr of Columbia	5.73	0.76	2.94	6.31	0.93	3.25	5.37	0.66	2.75
Florida	7.99	1.00	5.57	10.09	1.59	7.03	5.00	0.39	3.47
Georgia	7.46	1.12	5.22	9.17	1.69	6.42	5.87	0.69	4.13
Idaho	7.32	0.44	3.88	9.89	0.80	5.23	6.94	0.39	3.65
Illinois	5.70	0.45	4.12	7.95	0.88	5.75	4.65	0.30	3.36
Indiana	6.39	0.34	3.74	8.96	0.66	5.26	5.04	0.21	2.94
Iowa	6.06	0.18	2.72	8.92	0.39	3.99	6.82	0.23	3.07
Kansas	6.19	0.32	3.74	8.01	0.54	4.83	6.03	0.30	3.66
Kentucky	8.86	1.39	6.43	10.14	1.82	7.37	6.68	0.79	4.85
Louisiana	7.88	0.86	5.26	11.11	1.71	7.42	8.83	1.08	5.88
Maine	6.79	0.52	3.61	8.38	0.80	4.45	3.74	0.16	1.97
Maryland	6.31	0.81	4.94	7.76	1.22	6.09	5.41	0.59	4.22
Massachusetts	5.22	0.36	3.19	7.51	0.74	4.61	5.34	0.38	3.29
Michigan	6.56	0.42	3.89	11.18	1.21	6.65	7.52	0.55	4.47
Minnesota	5.32	0.26	2.99	7.64	0.53	4.30	5.49	0.27	3.11
Mississippi	9.08	1.38	6.48	11.32	2.15	8.06	10.51	1.85	7.48
Missouri	7.85	0.80	5.35	10.06	1.31	6.84	7.58	0.74	5.14
Montana	6.08	0.33	3.43	8.10	0.58	4.53	5.08	0.23	2.83
Nebraska	5.82	0.20	3.43	7.77	0.36	4.63	5.49	0.18	3.24
Nevada	6.82	0.43	3.35	10.83	1.09	5.32	8.57	0.68	4.20
New Hampshire	5.63	0.24	2.99	9.35	0.67	4.96	5.79	0.25	3.08
New Jersey	6.00	0.48	3.79	8.09	0.87	5.11	4.78	0.30	3.01
New Mexico	6.87	0.88	5.03	9.97	1.85	7.33	5.43	0.55	3.99
New York	5.66	0.41	4.08	7.93	0.81	5.73	5.88	0.45	4.26
North Carolina	7.20	0.75	4.72	8.66	1.09	5.68	7.85	0.90	5.14
North Dakota	5.92	0.24	3.37	8.91	0.54	5.08	6.29	0.27	3.59
Ohio	7.08	0.62	4.92	10.49	1.37	7.29	6.15	0.47	4.27
Oklahoma	7.57	0.65	4.18	10.32	1.20	5.74	6.84	0.53	3.80
Oregon	6.23	0.34	3.38	9.33	0.77	5.07	6.44	0.37	3.47
Pennsylvania	6.43	0.48	4.10	8.95	0.92	5.73	5.95	0.41	3.82
Rhode Island	5.20	0.29	2.49	7.13	0.55	3.41	6.11	0.40	2.90
South Carolina	7.47	0.89	5.14	8.92	1.26	6.13	6.74	0.72	4.62
South Dakota	6.96	0.54	4.36	8.95	0.89	5.62	5.53	0.34	3.46
Tennessee	8.86	1.22	7.04	9.47	1.39	7.53	6.67	0.69	5.31
Texas	9.12	1.62	9.25	10.88	2.31	11.06	5.78	0.65	5.88
Utah	6.07	0.55	5.58	8.93	1.19	8.20	6.80	0.69	6.27
Vermont	5.21	0.18	2.46	7.61	0.38	3.59	5.49	0.20	2.56
Virginia	6.75	0.97	5.14	7.79	1.28	5.93	4.49	0.43	3.42
Washington	6.28	0.69	4.27	8.52	1.26	5.81	6.11	0.65	4.16
West Virginia	10.68	1.34	6.52	13.55	2.15	8.32	7.19	0.61	4.41
Wisconsin	4.94	0.18	3.04	7.68	0.44	4.72	4.69	0.17	2.88
Wyoming	6.66	0.32	2.99	8.22	0.48	3.68	5.83	0.24	2.62
Alaska	7.01	0.69	4.04	8.96	1.13	5.17	6.08	0.52	3.51
Hawaii	6.72	0.20	1.96	8.46	0.32	2.44	7.12	0.22	2.07
US	6.89	2.35	11.61	9.26	2.96	13.42	6.27	2.10	11.43

## Conclusion

Geographic variations in the prevalence of chronic disease partly reflect the demographic composition of area populations. However, prevalence variations may also show distinct geographic 'contextual' effects that are differentiated between ethnic and other demographic categories. Studies of cardiovascular disease in the US have found major geographic variations that do not seem to be explicable by area demography alone.

The present study has demonstrated by formal modelling methods applied to BRFSS data that improved explanation is obtained by allowing for distinct geographic effects (for counties and states) and for interaction between geographic and person variables. There are significant spatial effects (e.g. county poverty effects, state residual effects) after adjusting for CVD gradients over person level variables, namely age, education, ethnicity.

This has direct implications for an appropriate methodology to estimate prevalence at small area level, with the focus here being ZIP Code Tabulation Areas. Thus – on the basis of the model estimates in the above analysis – prevalence estimates for a ZCTA need to reflect its region of location (e.g. in a South East state as opposed to a northern or mountain state) and the poverty level of the county containing it.

In methodological terms, this paper is distinct in using a log link multilevel binary regression model that takes account of both person level risk factors and the spatial context for a major chronic disease. The use of a log link allows straightforward inferences on relative risks and potentially allows the incorporation into the model of cumulative prior evidence (e.g. on relative CVD risks over ethnic groups). Statewide contextual effects have been represented by a structured random effect, that allows for spatial correlation in unobserved risk factors but also extends to include spatially isolated areas (see Appendix 2). In an extended model (model 3) state random effects are differentiated by ethnic group, reflecting evidence from other sources that ethnic relativities are not constant geographically.

Variations and extensions to the models presented above are possible. One option is state or county averages in the person level variables such as ethnicity and education level (e.g. county percent black or county percent college graduates). This has been proposed as a way of measuring contextual effects [34], though there is likely to be a positive correlation with the already included county poverty rate. Another possibility would be a longitudinal analysis over a sequence of successive surveys, which can indicate whether gradients over person level risk factors are changing, or whether geographic variability is changing.

## Appendix 1 Formal statement of model

Let *C*_*i*_, *S*_*i *_and *U*_*i *_denote the county, state and (county level) rural-urban category of residence for respondent *i*. Also let {*x*_*i*_, *g*_*i*_, *e*_*i*_} denote the age, ethnicity and education level of respondent *i*. Then prevalence models are specific for gender *r*, and one may write prevalence model 3 (with ethnic-specific state effects) as

(A1.1) *y*_*ir *_~*Bin*(1,*p*_*ir*_)

(A1.2) *log*(*p*_*ir*_) = *α*_*r *_+ *β*_*r*_[*g*_*i*_] + *η*_*r*_[*e*_*i*_] + *γ*_*r*_[*g*_*i*_, *x*_*i*_] + *κ*_*r*_*Pov*[*C*_*i*_] + *δ*_*r*_[*U*_*i*_] + *w*_*r*_[*S*_*i*_, *g*_*i*_],

where *Bin*(*n, p*) denotes the binomial density, the parameters {*α*, *β*, *δ*, *η*, *κ *} are fixed effects, and the parameters {*γ*,*w*} are random. This model is run separately for males and females.

Since the parameters operate on the log relative risk scale, state level relative risks by ethnic group *ρ*_*rsg *_(after controlling for known person and county attributes) may be obtained by exponentiating the state effect, namely

(A1.3) *ρ*_*rsg *_= *exp*(*w*_*rsg*_).

Thus excess risk or unduly low risk may reflect geographic variations in prevalence that remain even after the impact of a range of important person and county attributes has been allowed for. Excess risk can be defined in terms of the 95% estimation interval for *ρ*_*rsg *_being confined to values above 1.

The baseline model 1 (with person level risk factors only) is

(A1.4) *log*(*p*_*ir*_) = *α*_*r *_+ *β*_*r*_[*g*_*i*_] + *η*_*r*_[*e*_*i*_] + *γ*_*r*_[*g*_*i*_,*x*_*i*_].

The intermediate model (model 2), including county regression terms, and state random effects, but not including area-ethnicity interactions is

(A1.5) *log*(*p*_*ir*_) = *α*_*r *_+ *β*_*r*_[*g*_*i*_] + *η*_*r*_[*e*_*i*_] + *γ*_*r*_[*g*_*i*_,*x*_*i*_] + *κ*_*r*_*Pov*[*C*_*i*_] + *δ*_*r*_[*U*_*i*_] + *w*_*r*_[*S*_*i*_].

Thus the unobserved state effects are assumed to be equal across ethnic groups.

For the unknown fixed effects parameters, namely {*α*_*r*_, *β*_*rg*_, *η*_*re*_} in model 1, and {*α*_*r*_, *β*_*rg*_, *η*_*re*_, *κ*_*r*_, *δ*_*ru*_} in models 2 and 3, diffuse normal priors with mean zero and variance 1000 are adopted. Corner constraints are used for the *β*_*rg*_, *η*_*re *_and *δ*_*ru *_parameters for identifiability, namely *β*_*r*1 _= *η*_*r*1 _= *δ*_*r*1 _= 0. To pool strength across the age pro les of different ethnic groups, a first order random walk prior is used for the *G*-dimensional vector *γ*_*rx *_= (*γ*_*r*1*x*_,.., *γ*_*rGx*_), *x *= 1,.., *X *of age effects across *G *ethnic groups. This has conditional form

(A1.6) $$\gamma_{rx}\sim N_{G}(\gamma_{r,x-1},\Omega_{r}^{-1}),$$

where the *G *× *G *matrix $\Omega_{r}^{-1}$ represents covariation between age mortality profiles of ethnic groups. The precision (inverse covariance) matrices Ω_*r *_are assigned a Wishart prior with identity scale matrix and *G *degrees of freedom, namely Ω_*r *_~ *Wish*(*I*,*G*).

## Appendix 2 State random effects

The 51 states in the model are the mainland US states (*k *= 1,.., 49) arranged alphabetically (Alabama to Wyoming, including the District of Columbia), together with Alaska and Hawaii (*k *= 50, 51). The presence of these two spatially isolated states complicates applications of standard approaches for spatially correlated effects, at least those based on a spatial contiguity matrix. It would still be possible to use a spatial model based on interstate distances, but this means that a spatial decay function in distance has to be specified and its parameters estimated. Here we follow the most common approach to spatial clustering, based on contiguity of areas, with a spatial effect that "should describe the fact that areas close to each other tend to behave similarly" [26].

One option that brings in all 51 states would be to follow the convolution approach of Besag et al [35] and assume there are two effects, one of which follows a conditional autoregressive scheme and applies only to the mainland states (*k *= 1,.., 49), while the other effect, applying to all 51 states is unstructured in the sense of not incorporating spatial structure.

Thus for states *k *= 1, 49 the total state effect would be

(A2.1) *h*_*k *_+ *w*_*k*_,

where *h*_*k *_represents spatially unstructured heterogeneity, and *w*_*k *_represents a conditional autoregressive scheme based on contiguity. The suffix *r *for gender is omitted for simplicity. Specified conditionally on effects *w*_[-*k*] _in the remaining 48 states, one has for mainland states k = 1,..,49

(A2.2) *p*(*w*_*k*_|*w*_[-*k*]_) ~ *N*(*W*_*k*_, *τ*_*w*_/*L*_*k*_),   *k *= 1,.., 49

where *τ*_*w *_is a variance parameter, *L*_*k *_is the number of states adjacent to state *k*, and *W*_*k *_is the average of *w*_*m *_over states *m *= 1,.., *L*_*k *_adjacent to state *k*. For example, *W*_1 _(for Alabama) would be an average of the four *w *effects for the contiguous states, Mississippi, Georgia, Florida and Tennessee. The prior for the *h*_*k *_would be over all 51 states, rather than the mainland 49 states, and typically specified as

(A2.3) *h*_*k *_~ *N*(0,*τ *_*h*_)   *k *= 1,.., 51,

where *τ*_*h *_is a variance parameter. Under this convolution approach, for states 50 and 51 (Alaska and Hawaii) the state effect would consist of *h*_*k *_only.

While this approach is an option when a collection of areas includes spatial isolates, it is not used here. The problems that occur with the model (*A*2.1) include identifiability, since only the total *h*_*k *_+ *w*_*k *_is identified by the data, and the heavy (i.e. non-parsimonious) parameterisation. Leroux et al [36] propose an alternative more parsimonious model that uses a single random effect, with a conditional form

(A2.4) $$w_{k}|w_{\left[-k\right]}\sim N(\frac{\lambda }{1-\lambda +\lambda L_{k}}\sum_{m\sim k}w_{m},\frac{\tau_{w}}{1-\lambda +\lambda L_{k}}).$$

where *m *~ *k *denotes states *m *adjacent to state *k*. This reduces to a purely spatial model, as in (*A*2.2), when *λ *= 1 and to pure heterogeneity (i.e. no spatial clustering) when *λ *= 0. The *λ *parameter can be estimated and provides a measure of spatial dependence actually present in the data.

Congdon [25] extends model (*A*2.4) to allow the spatial dependence parameters to vary by area, and a version of such an approach is used in the CVD prevalence modelling here. This extension allows spatial dependence to vary over sub-regions of the total region or nation being considered, and also allows for spatial outliers, distinct from their neighbours in terms of outcome level such as disease risk. Outliers would have relatively low *λ*_*k *_values, since spatial pooling (towards the neighbourhood average) is contra-indicated by the disparity between an area's risk and that of its neighbours. By contrast, areas surrounded by areas with similar levels of the outcome would have relatively high *λ*_*k *_values, since spatial pooling (towards the neighbourhood average) is supported by the data.

The conditional specification now takes the form

(A2.5) $$w_{k}|w_{\left[-k\right]}\sim N(\frac{\lambda_{k}}{1-\lambda_{k}+\lambda_{k}L_{k}}\sum_{m\sim k}\lambda_{m}w_{m},\frac{\tau_{w}}{1-\lambda_{k}+\lambda_{k}L_{k}}).$$

This model for spatial effects adapts to spatial outliers by taking *λ*_*k *_= 0, so that for the subset of areas which are not connected to other areas one has

(A2.6) *w*_*k *_~ *N*(0, *τ*_*w*_).

This approach extends to a multivariate random effect *w*_*k *_= (*w*_*k*1_,.., *w*_*kG*_) for *G *ethnic groups. With a uniform value of *λ *over areas the conditional mean under the Leroux et al [36] model is

(A2.7) $$E(w_{kg}|w_{\left[-k\right]})=\frac{\lambda }{\left[1-\lambda +\lambda L_{k}\right]}\sum_{m\sim k}w_{mg},$$

with inverse dispersion matrix (precision matrix)

(A2.8) *Prec*(*w*_*k*_|*w*_[-*k*]_) = [1 - *λ *+ *λL*_*k*_]Φ,

where Φ is a symmetric matrix of dimension *G*. Allowing for varying spatial dependence over the entire region/nation being considered, one has

(A2.9) $$\begin{array}{l} E(w_{kg}|w_{\left[-k\right]})=\frac{\lambda_{k}}{\left[1-\lambda_{k}+\lambda_{k}L_{k}\right]}\sum_{m\sim k}\lambda_{m}w_{mg},\\ Prec(w_{k}|w_{\left[-k\right]})=[1-\lambda_{k}+\lambda_{k}L_{k}]\Phi . \end{array}$$

In the application of (*A*2.5) in model 2, it is assumed that 1/*τ*_*w *_is gamma distributed a priori, namely 1/*τ*_*w*_*~Ga*(1, 0.001). This is approximately equivalent to assuming 1/*τ*_*w *_to be uniformly distributed while constrained to positive values. Such a choice of gamma prior for 1/*τ*_*w *_follows the strategy of studies such as [35] and [37]. In the application of (*A*2.9) in model 3, it is assumed that Φ is Wishart distributed, with *G *degrees of freedom and an identity scale matrix.

The varying spatial parameters in models 2 and 3 are assumed to be beta distributed

*λ*_*k *_~ *Be*(*ν*_1_, *ν*_2_)

where *ν*_1 _and *ν*_2 _are positive quantities equal to or exceeding 0.5. Thus *ν*_1 _= *ν*_2 _= 1 corresponds to a diffuse uniform prior *λ*_*k *_~ *U*(0, 1), while more informative priors are obtained for *ν*_1 _> 1 and *ν*_2 _> 1. A baseline is provided when *ν*_1 _= *ν*_2 _= 0.5, equivalent to a prior sample size of 1. It is assumed that *ν*_1 _~ *U*(0.5, 5) and *ν*_2 _~ *U*(0.5, 5). The average value of the *λ*_*k *_over all contiguous states can be obtained as

*λ*_*a *_= *ν*_1_/(*ν*_1 _+ *ν*_2_).

## Competing interests

The author declares that they have no competing interests.
